# Diphtheria

**Published:** 1878-01

**Authors:** 


### The Bistoury.

ELMIRA, N. Y., JANUARY, 1878.

THE BISTOURY is published Quarterly, upon the 1st of April, July, October and January, at Fifty Cents a year in advance.

### DIPHTHERIA.

The following is condensed from the elaborate article on Diphtheria, by Oertel, in Ziemssen's Cyclopædia of the Practice of Medicine:

The treatment is divided into—I. Local;—II. General;—and III. Treatment of the Sequelæ. Lack of space will prevent a reviewal of the treatment of complications and sequelæ.

In the treatment of the local affection we are, 1st. To treat the inflammation, depending on the infection and its immediate results; endeavoring to stop the spread of the process and the passing of the disease into a severer form than that in which it came under treatment; to bring about a retrogression of the inflammatory process with removal of the pseudo-membranes and necrosed tissues. 2d. To prevent septic disease, and a general poisoning of the system, by the early and most complete removal possible, from the tissues, of all substances which cause the disease; by the arrest of the decomposition going on in the products of the disease; by the most general disinfection possible, and especially the cleansing of the mouth and throat; and, finally, the prevention of any further entrance of micrococci and products of decomposition from the surfaces of the diseased tissues.

In this disease general *blood-letting* is not to be thought of, and local blood-letting proves of no use, while even a slight loss of blood will only hasten the failure of strength, which is imminent without this. The inflammation is caused and kept up by infectious substances, by the luxuriant growth of micrococci upon which *cold* has no destructive effect, for specimens in water exposed for twenty-four hours to a temperature 4° below zero, Fahr., showed themselves capable of motion and propagation after the melting of the ice. Again, when the membranes become detached by the unaided efforts of Nature, it is invariably done through suppuration; the entrance of micrococci and putrefying substances is prevented by a thick, impermeable layer of pus-corpuscles, which form a separating stratum between the surface of the mucous and the false membranes. The removal of dangerous matters is also accomplished by means of suppuration; hence the hastening of this process means the early removal of the membrane and slight general poisoning of the system. Under the application of ice we find that, the fibrinous secretion goes constantly on; the formation of a purulent layer of demarcation and spontaneous detachment does not occur, and the disease is not retarded in its course.

If we attempt the *mechanical* detachment of the membrane while it is still adherent, the surface of the mucous membrane is wounded, as shown by bleeding points where its capillaries have been torn, thus affording an easier entrance of the parasites and products of decomposition into the tissues. The membrane, also, is rapidly reproduced, spreading over a greater extent, and the vast majority of patients, where this procedure is resorted to, succumb to the infection of the general system.

By *cauterization* it is impossible to completely annihilate the contagious material. The result of even the most prudent cauterization is a certain degree of mechanical violence which, with the chemical irritation, causes an increased inflammation, furnishing a much more favorable soil for the growth and spread of the poison.

Attempts to dissolve the false membranes by *chemical* means has not the least influence on the mucous membrane as long as the inflammation itself has not subsided. A new fibrinous exudation takes place, a second and even a third membrane forms without the treatment gaining any advance upon the disease, and the danger of systemic infection is as little diminished as by the mechanical detachment. Therefore this procedure is uncalled for, unless it is a question of treating a mechanical closure of the air passages, or of averting the danger of threatening suffocation.

The use of *astringents* increases and keeps up the inflammation, while it fails to diminish the fibrinous exudation. Neither does it effect the detachment of the pseudo-membranes and destruction of the masses of the micrococcus. Nor have these remedies any curative action, since statistics prove them to be completely useless in every important case.

In contrast to all these methods of limiting the further progress of diphtheria, is the effort which has been made to excite energetically, a rapid and abundant production of pus. Oertel endeavored to solve the problem by the employment of moist warmth, in the form of hot vapor, by means of which a temperature of from 113° to 122° Fahr.

was kept up for a considerable time in the mouth of the patient. The first appearances observed as a result of the operation of hot vapor are noticable in from twelve to eighteen hours; but these effects will be developed more slowly, if a considerable fibrinous exudation, with partial decomposition of the membranes has taken place. The margins of the deposits at the first glance seem enlarged; the operation of hot vapor, however, has been to induce a considerable excretion of pus-corpuscles, and these have infiltrated the epithelium. Under longer continued action of the hot steam, soon no further enlargement of the patches will be noticed; the false membranes become gradually thicker and raised up from the mucous membrane; their color becomes more of a yellowish or of a dirty gray, and their surfaces wrinkled and uneven, while the redness of the adjacent mucous membrane also fades and the swelling disappears. After some days we obtain, with the necessary amount of suppuration, a complete detachment of the pseudo-membranes. In the application of the hot vapor we may use an apparatus expressly made for such purposes, or an ordinary broad pot, with boiling water or infusion of mallows, from which the vapor is conducted through a suitable funnel, as hot and abundant as possible, into the mouth of the patient. The length and frequency of the inhalations must be determined by the degree of infection. If we aim at producing a rapid and free suppuration, the inhalations must be practiced as often and as long as possible, in quarter-hour sittings every half hour, and on the first and second days, three or four hours sleep must suffice for the patient. Later on, when the membranes have been partially cast off, as well as in lighter cases, hourly sittings of fifteen minutes, suffice, and a longer time can be allowed for the night's rest. Even after the membrane has separated completely, so long as a secretion of pus is perceived at the diseased places, occasional inhalations should be practiced, and these are to be suspended only after the cleansing of the mouth and throat is complete.

2. Prevention of septic disease and general systemic poisoning.

We possess no method of completely disinfecting the diseased organs. Diphtheritic micrococci after freezing and thawing in water, retain their mobility and increase in numbers. A similar result is obtained after allowing them to be acted upon by a boiling heat, or by solutions of sulphate of quinine, alum or chlorate of potash. Chloroform and ether-water developed only slight disinfecting properties. The most favorable results were obtained from freshly prepared chlorine-water, from fifteen to thirty parts to one hundred of distilled water; from a solution of carbolic acid, two to five grains to the ounce; from a solution of permanganate of potash, one to three grains to the ounce, by means of which and also of alcohol, of 96°, the vitality and multiplication of the vegetable organisms was completely done away with. As these substances are not suitable for inhalation, they will be most judiciously used as gargles, or, in the case of small children, we must seek to cleanse the mouth and throat by the use of the syringe.

But, as these fluids only occasionally bathe the mouth and throat, a complete destruction of the masses of micrococus cannot be effected, for they grow in the thick deposits and may have already entered into the tissues of the mucous membrane, and into the lymphatic vessels. But if we induce an active cell-formation the micrococci are washed away with the pus; or in case of a thick layer of fungus, by a suppurative process of demarcation.

To set up a rapid and abundant suppuration will then form one of the first indications of our present task. The energetic use of hot vapor will accomplish this agreeably to nature, and the longer the disease has lasted and the greater the extent of the exudation, so much the more must we push the use of the vapor, the highest possible temperature being used; at the same time the mouth and throat must be syringed out *every hour* with diluted alcohol or chlorine water, or solutions of carbolic acid and permanganate of potash.

The *General Treatment* is based upon the same principles as in other infectious diseases. We have therefore:

1. To diminish and ward off the constitutional disturbance, fever, and complications likely to arise.

2. To increase the patients power of resistance, in order that he may be able to live through the stage of reaction.

Cooling and antifebrile remedies should be given during the commencement of the sickness, and diarrhœa, if it exists, must be energetically combatted. The strength is to be husbanded by every possible means. The diet should consist of milk, concentrated meat broths, soups to which the yolk of egg has been added, &c. If the pulse becomes frequent and lacking in force, large doses of quinine, the tincture of chloride of iron, or the stronger wines are called for, and even large doses of cognac are well borne where the hearts action is very weak and there are evidences of septic poisoning.

